# Mental health services during the first wave of the COVID-19 pandemic in Europe: Results from the EPA Ambassadors Survey and implications for clinical practice

**DOI:** 10.1192/j.eurpsy.2021.2215

**Published:** 2021-06-09

**Authors:** Martina Rojnic Kuzman, Simavi Vahip, Andrea Fiorillo, Julian Beezhold, Mariana Pinto da Costa, Oleg Skugarevsky, Geert Dom, Izet Pajevic, Alma Mihaljevic Peles, Pavel Mohr, Anne Kleinberg, Eka Chkonia, Judit Balazs, William Flannery, Ramune Mazaliauskiene, Jana Chihai, Jerzy Samochowiec, Doina Cozman, Goran Mihajlovic, Lubomira Izakova, Celso Arango, Philip Goorwod

**Affiliations:** 1 Zagreb School of Medicine, Zagreb University Hospital Centre, Zagreb, Croatia; 2 Department of Psychiatry, Ege University Medicine Faculty, Affective Disorders Unit, Izmir, Turkey; 3 Department of Psychiatry, University of Campania “L. Vanvitelli”, Naples, Italy; 4 Norwich Medical School, University of East Anglia, Norwich, United Kingdom; 5 Institute of Psychiatry, Psychology & Neuroscience, King’s College London, London, United Kingdom; 6 Institute of Biomedical Sciences Abel Salazar (ICBAS), University of Porto, Porto, Portugal; 7 Psychiatry & Medical Psychology Department, Belarusian Psychiatric Association, Belarusian State Medical University, Minsk, Belarus; 8 Belgian Professional Association of Medical Specialists in Psychiatry, Collaborative Antwerp Psychiatric Research Institute (CAPRI), University of Antwerp (UAntwerp), Antwerp, Belgium; 9 Department of Psychiatry University Clinical Center Tuzla, School of Medicine University of Tuzla, Bosnia and Herzegovina Psychiatric Association of Bosnia-Herzegovina, Tuzla, Bosnia-Herzegovina; 10 Croatian Psychiatric Association, Zagreb, Croatia; 11 Czech Psychiatric Association, National Institute of Mental Health, Klecany, Czechia; 12 Third Faculty of Medicine, Charles University, Prague, Czech Republic; 13 Tallinn Children Hospital Children Mental Health, Tallinn, Estonia; 14 Estonian Psychiatric Association, Centre Tartu University Psychiatry Clinic, Tartu Estonia; 15 Society of Georgian Psychiatrists, Tbilisi State Medical University, Tbilisi, Georgia; 16 Department of Developmental and Clinical Child Psychology, Institute of Psychology, Eötvös Loránd University, Budapest, Hungary; 17 Hungarian Psychiatric Association, Bjørknes University College, Oslo, Norway; 18 Department of Adult Psychiatry, College of Psychiatrists of Ireland, Mater Misericordiae University Hospital, Dublin, Ireland; 19 Lithuanian Psychiatric Association, Lithuanian Health Sciences university, Psychiatric Clinic, Lithuanian Health Sciences university Kaunas hospital, Kaunas, Lithuania; 20 Society of Psychiatrists, Narcologists, Psychotherapists and Clinical Psychologists from Republic of Moldova, Department of Psychiatry, Narcology, Medical Psychology State Medical and Pharmaceutical University “Nicolae Testemitanu” from Republic of Moldova, Kishinev, Moldova; 21 Polish Psychiatric Association, Department of Psychiatry, Pomeranian Medical University in Szczecin Poland, Szczecin, Poland; 22 Romanian Association of Psychiatry and Psychotherapy, University of Medicine and Pharmacy Iuliu Hatieganu, Cluj-Napoca, Romania; 23 Department of Psychiatry, Faculty of Medical Science, University of Kragujevac, Kragujevac, Serbia; 24 Slovak Psychiatric Association, Department of Psychiatry, Faculty of Medicine, Comenius University in Bratislava, Bratislava, Slovakia; 25 Department of Child and Adolescent Psychiatry, Institute of Psychiatry and Mental Health, Hospital General Universitario Gregorio Marañón, IiSGM, CIBERSAM, School of Medicine, Universidad Complutense, Madrid, Spain; 26 INSERM, U1266 (Institute of Psychiatry and Neuroscience of Paris), Université de Paris, Paris, France; 27 CMME, GHU Paris Psychiatrie et Neurosciences, Hôpital Sainte-Anne, Paris, France

**Keywords:** COVID-19, mental health services, Europe

## Abstract

**Background:**

The COVID-19 pandemic caused an unprecedented worldwide crisis affecting several sectors, including health, social care, economy and society at large. The World Health Organisation has emphasized that mental health care should be considered as one of the core sectors within the overall COVID-19 health response. By March 2020, recommendations for the organization of mental health services across Europe have been developed by several national and international mental health professional associations.

**Methods:**

The European Psychiatric Association (EPA) surveyed a large European sample of psychiatrists, namely the “EPA Ambassadors”, on their clinical experience of the impact of COVID-19 pandemic on the treatment of psychiatric patients during the month of April 2020 in order to: a) identify and report the views and experiences of European psychiatrists; and b) represent and share these results with mental health policy makers at European level. Based on the recommendations issued by national psychiatric associations and on the results of our survey, we identified important organisational aspects of mental health care during the peak of the first wave of the COVID-19.

**Results:**

While most of the recommendations followed the same principles, significant differences between countries emerged in service delivery, mainly relating to referrals to outpatients and for inpatient admission, assessments and treatment for people with mental disorders. Compared to previous months, the mean number of patients treated by psychiatrists in outpatient settings halved in April 2020. In the same period, the number of mentally ill patients tested for, or developing, COVID-19 was low. In most of countries, traditional face-to-face visits were replaced by online remote consultations.

**Conclusions:**

Based on our findings we recommend: 1) to implement professional guidelines into practice and harmonize psychiatric clinical practice across Europe; 2) to monitor the treatment outcomes of patients with COVID-19 and pre-existing mental disorders; 3) to keep psychiatric services active by using all available options (for example telepsychiatry); 4) to increase communication and cooperation between different health care providers.

## Introduction

The COVID-19 pandemic has led to a worldwide crisis in a variety of sectors, including health, social welfare, and society at large. The World Health Organisation (WHO) declared that mental health services should be considered one of the essential health services to be maintained during the COVID-19 health crisis for different population levels [1], because the pandemic is associated with a significantly increased demand for mental health services [2,3]. For example, up to a third of persons who develop COVID-19 exhibit neuropsychiatric manifestations directly due to the infection itself [4] and significant numbers of patients are presenting with deterioration in their existing mental disorders, along with many with new anxiety and mood disorders triggered by the pandemic and its economic and social consequences [5–7]. People with chronic mental disorders have increased mortality risk factors, including smoking, metabolic syndrome and hypertension and may be at increased COVID-19 infection risk [8,9]. Importantly, frontline medical staff suffers from higher levels of burn out [10,11].

Apart from an increased demand for mental health services for different levels of population, the pandemic has also brought the need for fast and flexible adaptations in the organization of mental health services to respond to these increased demands. There was also a need to maintain mental healthcare for all persons currently treated for pre-existing mental health disorders, who need continuous and un-interrupted medical care, for example, regarding substitution therapy or long acting injectable medications. Moreover, psychiatric facilities with patients hospitalized due to chronic psychiatric conditions constituted particularly vulnerable units, due to greater potency of the virus spreading [12].

However, despite these requirements, during the first wave of the COVID-19 pandemic, psychiatric services were reduced to emergency care only in most countries, with the few remaining services which almost spontaneously shifted from the traditional face-to-face services to the remote services (from telephone services to the use of different video platforms), as the only possible option [13].

## Response of the (Inter)national Psychiatric Associations to COVID-19 Pandemics in March 2020

With high demands for mental health services together with a dramatic increase of the number of patients with COVID-19 requiring all health resources to be directed to the prevention of the pandemic and the organization of healthcare for COVID-19 patients, different solutions by national policy-makers were searched for within different countries.

In line with WHO recommendations [1], mental health national and international professional associations across Europe issued recommendations during the first peak period of COVID-19 pandemic (in March 2020) for the organization of mental health services. However, despite those efforts, disruption in mental health service delivery was observed in most countries [13].

According to the websites of the European and the World Psychiatric Associations, the majority of European national psychiatric associations provided a series of guidance documents (https://www.europsy.net/epa-resources-for-covid-19/ and https://www.wpanet.org/covid-19-resources?lang=de) which defined the basic organization of psychiatric care in their countries [14,15]. The topics of these documents, as well as the major recommendations included in those documents, were relatively similar across different professional associations, as well as across the guidelines issued by the organizations of patients and their families active in Europe (The Global Alliance of Mental Illness Advocacy Networks-Europe GAMIAN-Europe and EUFAMI). In general, and in line with the WHO recommendations [1], all stressed the need to maintain the care for persons with pre-existing mental healthcare and comply with epidemiological measures; all stressed psychosocial consequences of the restrictive public measures to general population and frontline medical workers, and all recommended organizational changes in the direction of offering remote instead of face-to-face services. These recommendations are summarized in Table 1.

**Table 1.** Recommendations on psychiatric care during the COVID-19 pandemic issued by European Psychiatric Associations in March 2020.

Topic	Major recommendation
Organization of care for persons without pre-existing mental disorders	
Psychosocial approach to persons in crisis, the effects of quarantine	Stress management for different population and for frontline medical workers working in COVID-19 units
Recommendations for medical professionals	Stress management for frontline medical workers working in COVID-19 units
Clinical management for patients with SARS-CoV-2 and mental health symptoms/problems	Recommendation for the use of psychopharmacology and therapeutic procedures; increased collaboration of frontline doctors and mental health professionals
Organization of care for persons with pre-existing mental disorders	
Prevention of COVID-19 in psychiatric facilities	Strict adherence to epidemiological measures in psychiatric facilities
Telepsychiatry	Replacement of traditional face-to-face visits with online visits
Clinical management for patients with pre-existing mental health problems	Recommendations for the use of medication and procedures requiring long term use (long acting medication, substitution therapy in addiction disorders)
Child and adolescent psychiatry	Protection of mental health of children and adolescent, recommendation for the prevention of domestic violence
Old age psychiatry	Identification of risk factors for severe forms of COVID-19, recommendation for specific treatment in old age population
Forensic psychiatry	Prevention of COVID-19 outbreak in facilities
Preventive/social psychiatry	
Call for transparent management Call for increasing solidarity, not the worry and fear Call for responsible news and programs in media Warning against possible discrimination towards psychiatric patients in COVID-19 wards	Decrease possible risk factors on mental health and wellbeing on the level of public health

Although the recommendations were intended for mental health professionals, other medical specialties, general population and policy-makers, these were generally issued by the national psychiatric associations. The narrow organizational aspect of psychiatric services during COVID-19 (definition of facilities, equipment, and staff responsible for the treatment of persons with COVID-19 and pre-existing mental health disorders) was described in several recommendations issued by the national associations, while in some countries the psychiatric associations provided the supporting documents issued by the national Ministry of Health. In a few countries (e.g., Greece, Turkey, and Croatia), recommendations on broader topics, such as measures to reduce the stigma associated with COVID-19 and mental health, were also developed. As a close collaboration of different stakeholders on a national level is needed to implement new recommendations, it is probable that these efforts produced different effects in different countries. Indeed, despite those efforts a disruption in the organization of mental health services in most countries was observed during the first wave of the pandemics [13]. Moreover, in some of the countries, this situation resulted in psychiatric departments being (temporarily) closed and transformed into COVID-19 wards [16], which may be expected considering the levels of stigma associated with psychiatry.

## Changes of Mental Health Services in Europe in April 2020

To assess psychiatric care throughout time periods, the European Psychiatric Association (EPA) approached psychiatrists working in Europe, who are either individual members of the EPA, attendees of the congresses of the EPA or the members of the EPA Council of National Associations member societies and early career psychiatrists (ECP). They were offered to become “EPA Ambassadors” and to participate in EPA surveys to rapidly obtain Europe-wide information regarding psychiatric care and services. The Ambassadors agreed to collect important information in their own country, to inform stakeholders at the European level, therefore facilitating faster actions and adaptations of mental health policies in Europe. The questionnaire focused on the organization of mental healthcare for patients with pre-existing mental health problems during the first wave European COVID-19 pandemic peak (April 2020) and included questions on the access to care (the estimation on the number of patients treated, estimation of the number of patients tested, and positive for COVID-19), modalities of visits (the estimation on the number of patients who were visited using remote services instead of face to face visits), the type of planned facilities for patients with mental health problems and COVID-19 and the level of satisfaction with the collaboration between different stakeholders in organization of mental healthcare. Questions also inquired data on sociodemographics (age, gender, duration of mental healthcare practice, position, type of work, and place of work).

Responses were collected from July to August 2020, using an online questionnaire. Psychiatrists who are either individual members of the EPA, attendees of the congresses of the EPA or the members of the EPA Council of National Associations member societies and ECP were invited to participate by sending them an invitation with a link to the online questionnaire. Responses were collected from July 2020 to August 2020. The survey was open to all clinicians in mental health, and in this regard, do not pretend to have any exhaustivity nor representativity. We initially sent an invitation email to the EPA list of past participants (who agreed to be contacted), which means around 10,000 e-mail addresses. We contacted all National Psychiatric Associations (*N* = 44) and the Council (ECP) early career psychiatrists (*N* = 1) who were asked to distribute the invitations among the members of their associations.

Nine hundred and forty responses were collected, with 857 participants practicing in Europe. Therefore, only responses from the 857 participants practicing in Europe were included in the final analyses. Among them, 56% of respondents were female (*N* = 480), 38.8% (*N* = 333) were below 40 years of age, whereas 28.9% (*N* = 419) were aged 41–60, and the rest (32.3%) were aged over 60. Most responders were psychiatrists (75.5%, *N* = 647) followed by trainees in psychiatry (13.3%, *N* = 117), other mental health professionals (including psychologists, social workers, and nurses; 5.7%, *N* = 49), and users of mental health services (0.8%, *N* = 7).

### Access to consultations with psychiatrists

We categorized countries to three regions according to EuroVoc.

1. Central and Eastern Europe: Albania, Armenia, Azerbaijan, Belarus, Bosnia and Herzegovina, Bulgaria, Croatia, Czech Republic, Estonia, Georgia, Hungary, Kyrgyzstan, Latvia, Lithuania, Macedonia, Moldova, Poland, Romania, Russian Federation, Serbia and Montenegro, Slovakia, and Ukraine.
2. Northern and Western Europe: Belgium, Denmark, Finland, France, Germany, Ireland, Netherlands, Slovenia, Sweden, Switzerland, and United Kingdom.
3. Southern Europe: Greece, Israel, Italy, Portugal, Spain, and Turkey.

Before the analyses, we weighed our sample according to the total number of psychiatrists working in each country, so that the structure of the sample had the same structure as the targeted population. We provide both crude and weighted estimates. For each region, we calculated the median and interquartile range (IQR) of number of patients before pandemic and during the April 2020, number tested, percentage tested out of all those seen during April, and the estimated percentage affected with SARS-CoV-2 out of all patients seen during April 2020. For the difference between the number of patients seen before the pandemic and during April 2020, we calculated the median of absolute differences (∆) with the 95% Bonett-Price confidence interval (CI), statistical significance of the differences using the Wilcoxon rank-sum (Mann–Whitney *U*) test, and the percentage change (∆%) relative to the number of patients before the pandemic. To test the significance of differences in medians between the three regions, we used the quantile regression. As the maximum missing data for any variable was ≤1%, we excluded these cases from specific analyses (pairwise deletion). We controlled for the inflation of false positive results caused by multiple testing by using the Benjamini–Hochberg procedure with a false positive rate set at FDR < 5%. We calculated all CIs at 95% level and set the two-tail significance rate at 0.05. We performed statistical data analyses using StataCorp 2019 (Stata Statistical Software: Release 16. College Station, TX: StataCorp LLC).

While 857 participants responded, 236 had no data on the number of patients before the pandemic, 12 more declared that they had zero patients before and during April as being tested or infected, and 1 participant declared no patients seen before the pandemic, nor during April. The final sample size was therefore 608, consisting only of participants who practice in Europe, and who had at least one patient monthly before the pandemic.

In the weighted total sample, the median number of patients seen monthly by one psychiatrist before the pandemic was 50 (IQR 30–100), and the difference between the three regions were not significant (Table 2). The median number was the same in Central and Eastern Europe than in Northern and Western Europe, the latter being used as the reference region (∆ = 0; 95% CI −38; 38; *p* > 0.999; FDR > 5%). In Southern Europe, the median number of patients was larger than in Northern and Western Europe, but the difference was not significant (∆ = 30; 95% CI −6; 66; *p* = 0.101; FDR > 5%). During April 2020 the number of patients decreased by 10 (95% CI −15; −5; *p* < 0.001; FDR < 5%), corresponding to 25% less median number of patients monthly. The relative decrease in the number of patients during April was significantly different between the three European regions. The decrease was the largest in Southern Europe (∆ = −42 compared to the Western Europe; 95% CI −64; −20; *p* < 0.001; FDR < 5%), but it was also significant in the Central and Eastern Europe (∆ = −25 compared to the Western Europe; 95% CI −28; −3; *p* = 0.025; FDR < 5%). We have not observed any significant difference between the three regions in the number of tested patients, the percentage tested out of all patients seen during April, and the percentage infected by SARS-CoV-2 out of all those seen during April (Table 2).

**Table 2.** Estimation of numbers of patients with mental health problems seen by psychiatrists per month before and during the pandemic in different countries.

	*n*	Before pandemic		During April 2020^a^		Difference in the number of patients during the first lockdown				Number tested		Percentage tested out of all in April		Percentage infected	
		*M*	(IQR)	*M*	(IQR)	∆	(95% CI)	*p*	∆%	*M*	(IQR)	*M*	(IQR)	*M*	(IQR)
Crude sample															
Whole Europe	608	80	(40–150)	40	(20–80)	−20	(−25; −15)	<0.001^b^	−40	3	(0–10)	7	(0–25)	1	(0–10)
Northern and Western	168	50	(30–100)	40	(20–70)	−5	(−10; −0)	0.044^b^	−13	2	(0–7)	4	(0–20)	1	(0–10)
Southern	197	100	(50–200)	45	(20–98)	−40	(−57; −23)	<0.001^b^	−54	4	(0–10)	7	(0–31)	3	(0–13)
Central and Eastern	243	80	(35–150)	30	(20–80)	−20	(−27; −13)	<0.001^b^	−40	3	(0–10)	10	(0–31)	0	(0–6)
Weighted sample^c^															
Whole Europe	608	50	(30–100)	40	(20–70)	−10	(−15; −5)	<0.001^b^	−25	3	(0–5)	7	(0–25)	0	(0–12)
Northern and Western	168	50	(30–100)	47	(20–65)	−8	(−18; 2)	0.110	−8	4	(0–5)	5	(0–17)	0	(0–12)
Southern	197	80	(40–150)	40	(15–80)	−30	(−42; −18)	<0.001^b^	−50	2	(0–10)	8	(0–33)	3	(0–10)
Central and Eastern	243	50	(25–100)	30	(15–70)	−10	(−14; −6)	<0.001^b^	−33	2	(0–10)	10	(0–40)	0	(0–13)

Data are presented as median number of patients if not stated otherwise.

Abbreviations: ∆, median of differences between the number of patients before pandemic and during April 2020; ∆%, median of relative differences in number of patients calculated as the number of patients (April—before)/before; CI, Bonett-Price confidence interval for median; IQR, interquartile range; n, number of participants; M, median; p, statistical significance of the absolute difference calculated using Wilcoxon rank-sum (Mann–Whitney *U*) test.

a There were 4 (0.7%) missing data for number of patients during April 2020; 5 (0.8%) for number tested; and 6 (1.0%) for number infected.

b FDR < 5%.

c Samples were weighted for the total number of psychiatrists in particular countries.

Although it is possible that this decrease in the number of visits resulted from public measures (such as physical distancing, quarantine and call for stay at home except in emergencies) which were in place in most countries during the study period and lower number of patients seeking psychiatric help at that time, this may also reflect the disruption of mental health services contrary to the recommendation of the WHO [1] and the recommendations of psychiatric associations [14,15]. Indeed, the WHO survey performed in 130 countries which used responses collated by national ministries of health, found that in most countries, disruption in the organization of outpatient and community mental health services was observed, while the hospital-based services remained fully open in around 70% of countries. Services for substance use disorders were the most affected. Specific interventions for old age psychiatry, promotion, and prevention services (including those for patients with drug addiction and for suicidal patients), psychotherapy and child and adolescent psychiatry were most disrupted [13].

Interestingly, the number of psychiatric patients being tested for COVID-19 was quite low as well as the number of positive cases, without any significant differences between the regions. (Table 2). While this may reflect a protective effect from social isolation of patients with severe psychiatric disorders, it may not be different from other groups who were aware that they may be at risk of severe COVID-19 forms (e.g., older adults, people with chronic morbidities, or cancer or compromised immune system). Thus, it is also possible that these figures increased over the following months. However, large population studies in the United States report contrary findings, with persons with pre-existing psychiatric disorders more likely to develop COVID-19 infection [9,17]. While the reasons for this finding are unclear, and in part also explained by the accessibility of the medical care and the overall living conditions and socioeconomic status for the persons with mental illness in the United States, it would be of interest to examine the dynamics of the rates of patients with psychiatric conditions who are exposed and infected with SARS-CoV-2 and develop COVID-19 over specific phases of pandemic, as it should be considered when planning the organization of healthcare for persons with pre-existing mental health problems.

### Replacement of face-to-face by remote consultations

**Figure 1.**
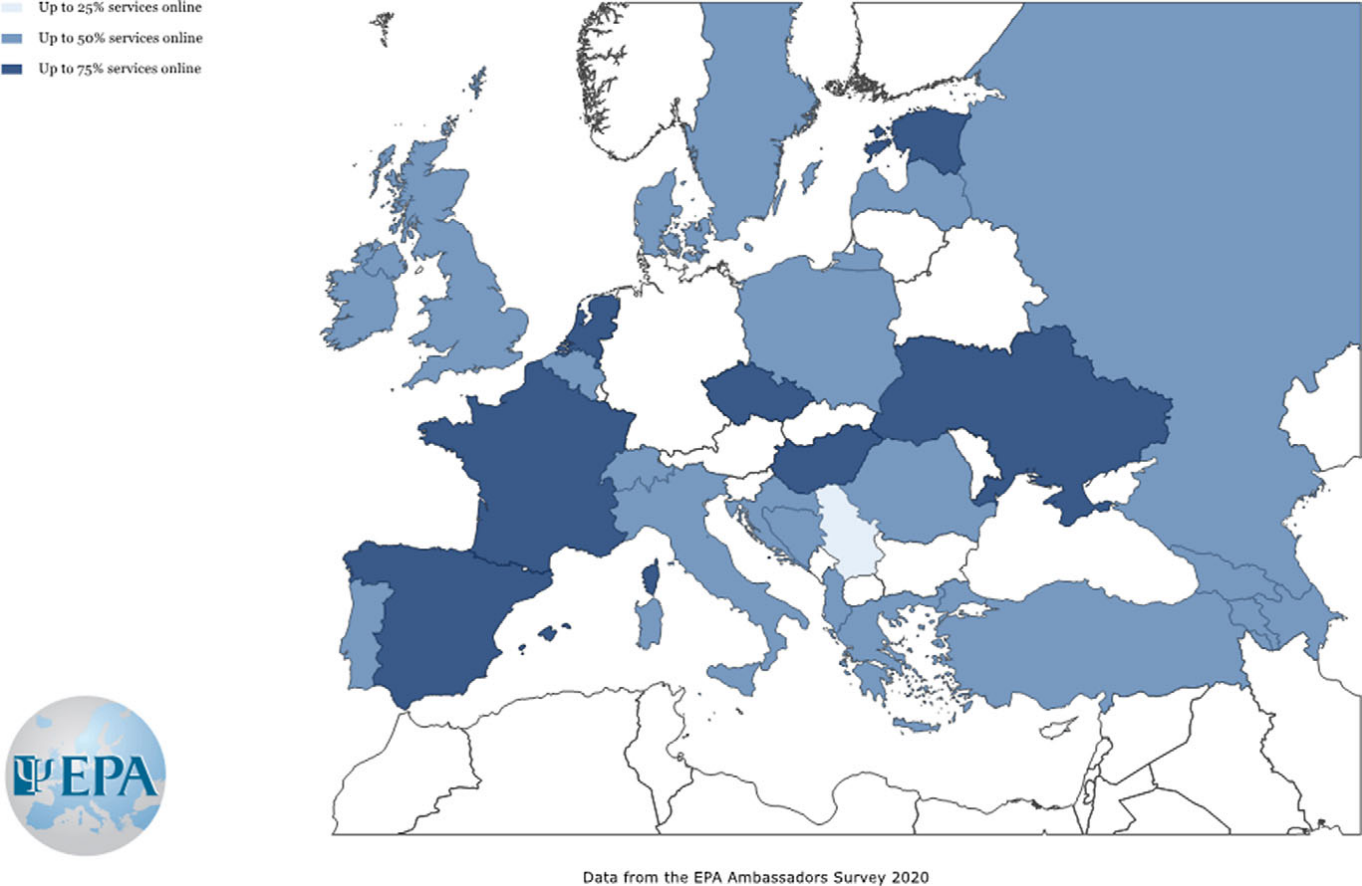
Estimation of the percentage of on-line services provided instead of face-to-face consultations by mental health professionals in April 2020 across European countries. Countries with a number of responses lower than 5 were not shown.

In line with the recommendation by the psychiatric associations, in most European countries, face-to-face has been replaced by online consultations, online consultation delivery ranging from 25% to more than 75% of all consultations. However, several differences between countries have been detected. In most of the countries, approximately 50% of services were offered online, but in others, especially Western countries, the percentage of telepsychiatry consultations was significantly higher (Figure 1). Although most national psychiatric associations recommended replacing the traditional face-to-face with online or remote consultations, it seems that most countries lacked infrastructure, information technology expertise or legal frameworks for rapid provision of telepsychiatry during the first wave pandemic peak, which may explain the variation of the degree of the implementation across countries.

### Organization of mental health services for patients

**Figure 2.**
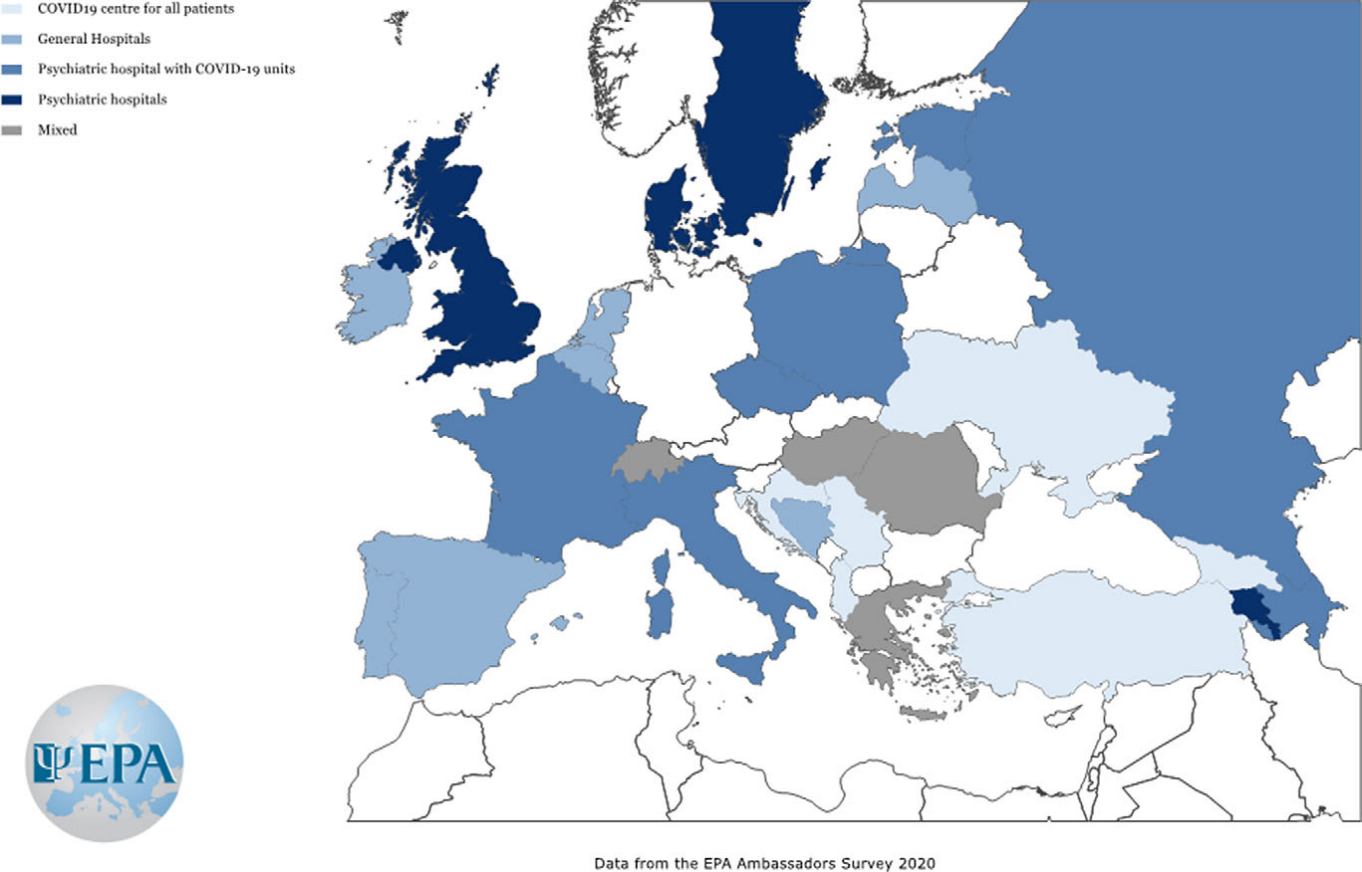
Predominant model of service for persons with pre-existing mental health problems infected with SARS-CoV-2 in April 2020 across European countries. Countries with a number of responses lower than 5 were not shown.

The re-organization of mental healthcare services for patients with mental disorders who were infected by SARS-CoV-2 was rather different across Europe. Indeed, we observed a high variation in hospital-based care for persons with COVID-19 and pre-existing mental disorders for these patients in the overall sample, ranging from “general hospitals” (28.8%), to “psychiatric hospitals” (22.1%), “COVID-19 wards specifically designed for psychiatric patients” (28.4%) and “COVID-19 wards for all patients” (20.7%) (Figure 2).

The creation of specialist units for patients with mental health problems who developed COVID-19 may reflect differences between mental health services in Europe in general. However, it may also reflect the fact that health services in general were struggling in March 2020, and possibly trying out different options, with rapid changes everywhere.

Considering that few national psychiatric associations issued recommendations on the organization of care for mentally ill patients who developed COVID-19, it is not known whether they were represented in local authorities (e.g., ministries) responsible for the organization of health services, and whether they contributed to the decision-making process.

Other factors including the stigma toward psychiatry may be also relevant to care delivery, for example, resulting in decreased availability of adequate healthcare for patients with mental illness [18]. This may be an argument for the organization of psychiatric care within specialist COVID-19 centers for all patients to guarantee an equal level of care, especially in countries where psychiatric facilities are less equipped with protective equipment, supply of oxygen, or have worse sanitary conditions.

This was recognized in some countries (e.g., Croatia) where the national psychiatric associations specifically tackled the problem of stigma in their March 2020 recommendations. It is noteworthy that some national psychiatric associations (e.g., Slovakia) clearly stated that adequate equipment and personnel in psychiatric facilities should be provided before psychiatric wards admitted patients with comorbid acute SARS-CoV-2 infection. This seems especially important considering the increased risk of patients with mental health problems for developing complicated forms of COVID-19 due to the presence of multiple risk factors [5].

In some countries such as France, the guidelines issued by professional associations supported the organization of COVID-19 units within psychiatric wards, which were adequately equipped with equipment and staff for the safe management of COVID-19, in concordance with the exiting reports from the literature [16].

However, organization of COVID-19 units within psychiatric hospitals may be problematic if these hospitals also include nearby wards for patients with chronic mental illnesses, forensic wards or old age psychiatric units; or with high levels of staff rotating between wards due to very high rate of spread of the SARS-CoV-2 virus once introduced [12,19].

Another option recommended by national associations (e.g., Turkey) includes different steps for psychiatric patients tested positive: giving priority to safe provision of the maximum possible quality psychiatric services using close and continuous consultation and liaison with COVID-19 services of general hospitals; and providing *at least one* fully equipped (physical needs, personal protective equipment, and staffing) COVID-19 psychiatry unit for each region of the country dedicated to care for acutely ill psychiatric COVID-19 positive patients. Due to established risk factors and high vulnerability to complicated forms of COVID-19 in older age or dementia patients, wards for old age psychiatry should be kept separate.

The impact of different models of service delivery is yet to be determined, therefore it is crucial that all facilities monitor the treatment outcomes for patients with COVID-19 and pre-existing mental disorders; and work to prevent the increased morbidity and mortality due to inequitable access to health services.

While service delivery for patients with acute mental health problems who develop COVID-19 may differ due to local circumstances, it is important that ethical principles are followed. Patients with psychiatric problems should receive the same level of healthcare for COVID-19, as persons without pre-existing mental disorders. This is especially important since reports from some countries (such as the United States) suggest a doubled mortality rate due to COVID-19 in patients with prior psychiatric disorders compared those without, even after controlling for medical comorbidities, thus highlighting the role of social, behavioral, and lifestyle factors in contributing to this profound inequality [9,17].

### Cooperation between mental health providers

**Figure 3.**
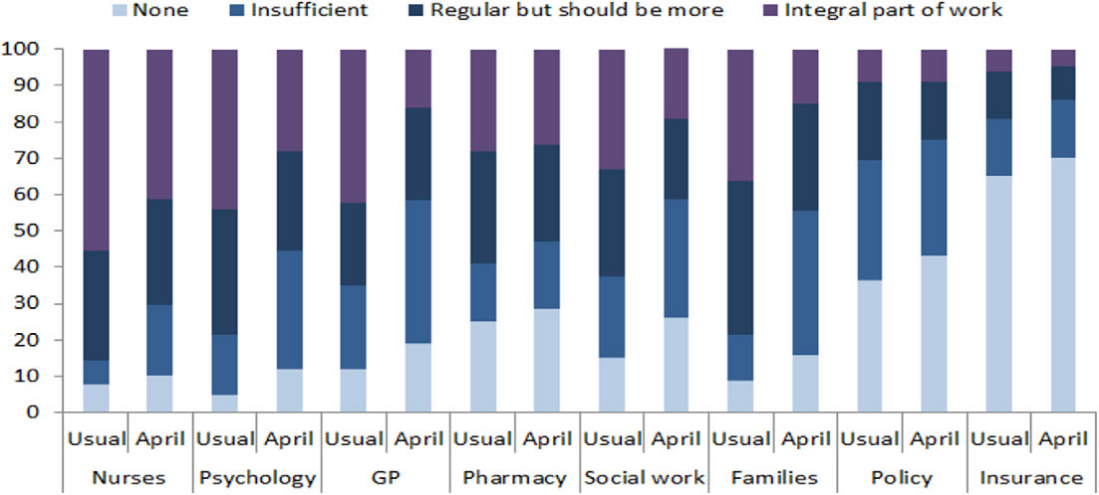
Cooperation between healthcare providers during April 2020 compared to the period before COVID-19 pandemic.

While an effective fight against pandemic COVID-19 required cooperation at different levels, most participants reported a drop in the quality of cooperation with nurses, general practitioners, psychologists, and family members with whom close collaboration had previously been a good and an integral part of work prior to the pandemic. On the other hand, no significant change was reported in the quality of cooperation with occupational therapists, physiotherapists, pharmacists, policy-makers, and insurance companies, reflecting the low levels of cooperation reported even before the COVID-19 pandemic (Figure 3).

This reduced or less effective cooperation with policy-makers may have adversely influenced effective psychiatric and health service delivery for persons with mental illness as well as the poor implementation of the recommendations issued by the psychiatric associations. For example, good cooperation with policy-makers and healthcare insurance companies may be crucial for introducing legislative and financial coverage for new ways of healthcare provision, such as telepsychiatry and remote working, or liaison psychiatric care within COVID-19 units [3]. Also, good cooperation with general practitioners, nurses and pharmacists may be critical for aspects of service delivery during pandemic times, for example, the organization of substitute therapy or change long-acting injectable medication especially during periods of lockdown and isolation. Finally, cooperation between psychiatrists and legal experts is needed to assure the human rights of patients [20].

Importantly, studies have shown that medical personnel experience increased amounts of insomnia, somatization, anxiety, and depression during a pandemic [21,22]. Thus, good cooperation between healthcare providers may also have a protective effect for clinicians working with COVID-19 patients, especially when it is difficult to predict a return to normality and the predicted consequences counted as years of life lost due to psychosocial consequences of COVID-19 mitigation measures [23,24]. Developing a support system at scale, for people affected by mental crises after various disasters, is a challenge for mental health services and requires engagement and support from all mental healthcare providers [3].

## Conclusions

National psychiatric associations responded rapidly to the first wave of the COVID-19 pandemic with most issuing recommendations already by March 2020 and were markedly consistent across Europe in terms of topics and overall recommendations. Nevertheless, contrary to the recommendations of the WHO [1] and the efforts of the national psychiatric associations [14,15], we detected significant changes in mental healthcare service delivery in April 2020, considering a significant drop in the number of outpatients visits across Europe countries, probably indicating the disruption of psychiatric services across Europe, and the effects on the lock down measures contributing to the decrease in the number of patients seeking psychiatric help on the other side.

With a significant reduction of in-person consultations mainly due to lockdown measures, most countries shifted, to varying degrees, toward online or remote psychiatric care as one of the only safe options available at the time. This might represent a significant barrier specifically for vulnerable, multi-problem patients often with less means to or competencies to access to digital communication. It is reasonable to expect that the use of telepsychiatry and remote consultation will increase further given the potential duration of the pandemic. As the equipment and legislation supporting the implementation of remote services may be lacking in many European countries, future efforts should focus on implementation research and implementation of the new remote services based on the models established in other European countries which proved effective but within specific local context in countries where this service is not fully implemented yet. There is a need for discussions on all aspects of the use of remote/telepsychiatry and finding ways to optimize implementation across countries [25]. When it comes for the hospital-based care for persons with COVID-19 and pre-existing mental disorders, we detected large differences between countries, in particular regarding access to services (with different level of access and types of consultations), assessment processes, and admission to hospital settings (specific wards for COVID-19 inpatients) for patients with psychiatric disorders. As the effect of these individual solutions on the delivery and quality of healthcare is not known, it would be of interest to continue to monitor the accessibility of and quality of psychiatric services (e.g., number of patients, waiting lists, type of services, type of personnel, equipment, etc.) based on the real life data and in specific national context, in order to make transparent decision on which services should continue and which should be re-organized.

According to our results, during the pandemics, most participants experienced a drop of the cooperation between different stakeholders including a poor cooperation with policy-makers. Thus, a better cooperation between different levels of healthcare providers must be guaranteed to assure an efficient and enduring provision of healthcare services and the implementation of recommendation by the national psychiatric associations in the real-world practice. As we expect the rise in mental health problems during and following the pandemic [21], since the infection with SARS-CoV-2 increases the risk of mental disorders in general population [22], medical workers [23,24], survivors of COVID-19 with postacute-COVID-19 [26], keens of the deceased from COVID-19 [27], it is reasonable to assume an increased demand for efficient mental health services. The cooperation of different stakeholders is necessary to assure the accessible and un-interrupted mental healthcare services under the new circumstances and the implementation of new types and modalities services, including remote services, possibly post-COVID-19 services, liaison services within COVID-19 units, and so on. The EPA, as the European umbrella psychiatric association representing individual members and 44 national psychiatric associations should play a role in monitoring and reporting real world data from the represented countries and actively participate with other European stakeholders in shaping the recommendations and standards for the organization of mental healthcare on the European level. However, given the high variation in the provision of psychiatric care across the countries observed in the survey, the EPA should facilitate the identification of country/region-specific elements contributing to this variation and facilitate wider implementations of good clinical practice throughout Europe in the direction of the improvement of the mental healthcare accessibility and quality of mental healthcare acknowledging the local context.

This survey had several limitations. The major limitation is certainly the lack of knowledge on the representativeness of the sample, because of the method of recruitment, as the survey was open to “all clinicians in mental health.” In this regard, we acknowledge that the sample does not have any exhaustivity nor representativity. Secondly, the overall number of participants is rather low, especially when it comes to the numbers in specific countries. Finally, the data obtained by the participants are relying on their estimations, based on their everyday clinical practice, rather than accurate number of cases based on national statistics.

Nevertheless, this survey presents data on the functionality of several important mental health services during the first wave of the COVID-19 pandemic in Europe. Although these data are changing inevitably due to the dynamics of the COVID-19 pandemics, they still present a valuable source of information on the development of the first response of mental health systems to the situation. This study also presents how different countries adapted to the disruption of the mental health services in pandemic in the first wave. While the mental health policies might have changed in the second and third waves, the adaptation to the first wave set out some of the important decisions to mental health services organization. Learning from this pandemic experience, from its start, seems crucial for the future. Secondly, we gathered data from many countries in Europe, including those from which we lack data when it comes to the organization of mental health in Europe. This information will be used by the EPA and Council of National Psychiatric Associations in coordinating the exchange of learning, facilitating wider implementation of good clinical practice throughout Europe and supporting all European authorities, organizations and mental health workers in their relevant needs.

## Data Availability

The data that support the findings of this study are openly available at www.europsy.net (https://www.europsy.net/app/uploads/2021/05/EPA-Ambassadors_Figure-1_-Estimation-of-the-percentage-of-on-line-services-provided.png; https://www.europsy.net/app/uploads/2021/05/EPA-Ambassadors_Figure-2_Predominant-model-of-service.png)
